# Volatile organic compounds (VOCs) as a rapid means for assessing the source of coprolites

**DOI:** 10.1016/j.isci.2023.106806

**Published:** 2023-05-04

**Authors:** Wanyue Zhao, Helen L. Whelton, John C. Blong, Lisa-Marie Shillito, Dennis L. Jenkins, Ian D. Bull

**Affiliations:** 1 Organic Geochemistry Unit, School of Chemistry, University of Bristol, Cantock’s Close, Bristol BS8 1TS, UK; 2 Department of Anthropology, Washington State University, College Hall, Pullman, WA 99164-4910, USA; 3 School of History, Classics and Archaeology, Armstrong Building, Newcastle University, Newcastle upon Tyne NE1 7RU, UK; 4 Museum of Natural and Cultural History, University of Oregon, Eugene, OR 97403, USA

**Keywords:** Biological sciences, Paleobiology, Archeology

## Abstract

The odor of rehydrated coprolites can be used as an informal means of fecal identification. To date, the analysis of volatiles emitted by coprolites from different sources has not been attempted, and the possibility of utilizing volatile organic compounds (VOCs) as fecal biomarkers unexplored. VOCs released by coprolites from the Paisley Caves, were analyzed using solid-phase microextraction (SPME), to assess the variance of results from different coprolites (carnivores, herbivores, or humans). Coprolites from carnivores can be clearly distinguished from those produced by herbivores and humans; these latter two are separated to a lesser degree. Eight discriminatory compounds differentiated between the coprolite sources, and their identities were verified using reference standards. Coprolites and their associated sediments could not be differentiated between using this method, suggesting leaching of VOCs into the burial matrix. This work provides an alternative, more rapid way to assess coprolite origin.

## Highlights

• We distinguished between coprolite sources based on headspace VOCs using SPME
• Carnivore coprolites are clearly differentiated from herbivore and human coprolites
• Herbivore and human coprolites can be distinguished to a lesser degree
• VOCs can rapidly establish the sources of morphologically defined coprolites

## Graphical abstract

**Figure:**
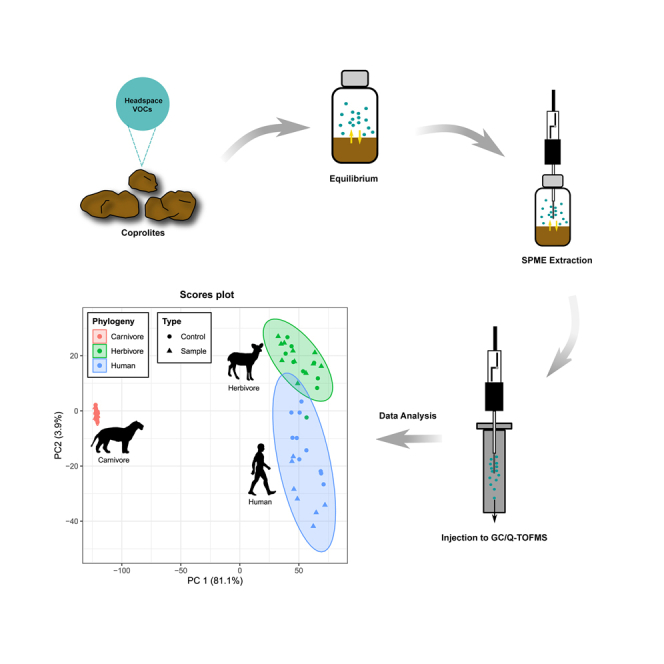

## Introduction

The accurate identification of fecal material, as either human or another animal, is essential for the correct interpretation of archaeological deposits. Ancient feces (coprolites) contain complex mixtures of lipids derived from the diet and physiological processes of the producer. Previous studies have targeted steroidal compounds (sterols and bile acids) using their relative distributions to inform whether deposits contain fecal material, and if so what the source may be.^1^ Specifically, 5β-stanols derived from sitosterol are found at high proportions in herbivore feces,^2^ whereas cholesterol-derived 5β-stanols (coprostanols) are found at high proportions in the feces of omnivores, with coprostanol being the predominant 5β-stanol in human feces.^3^ Ratios of the above stanols, in combination with bile acid distributions, enable identification of fecal material and its source.^1^ More recently, this approach has been refined to enable a more precise, species-level identification between a wider range of fecal sources such as donkey/cattle and sheep, goat/goose, horse, reindeer and dogs.^4^^,^^5^ Other lipid classes contained within coprolites, such as hydrocarbons and terpenoids, may also provide an indication of broad dietary inputs and can be used in conjunction with macro- and micro-morphological analyses to refine dietary information.^6^^,^^7^ However, a caveat of analyzing sterols and bile acids is the destructive nature of the analysis and the length of laboratory time required to prepare samples.

Solid-phase microextraction (SPME) of volatile organic compounds (VOCs) is a potential alternative approach to coprolite identification. VOCs are low molecular weight compounds, typically <300 Da, with high vapor pressures and low boiling points.^8^ The odor which occurs following rehydration of a coprolite has long been used for fecal identification and is suggestive of different VOC signatures between coprolites from different sources.^9^^,^^10^^,^^11^ VOC profiles associated with feces have also been adopted in clinical microbiology, where they have been shown to potentially reflect gut microbial metabolic activity, and may be adopted as biomarkers for specific diseases.^12^^,^^13^ Previous studies have proved that VOCs enable sensitive differentiation between soils receiving different organic amendments,^14^ and it is not much of a stretch to hypothesize that it may be possible to use VOCs to distinguish between coprolites that derive from different organisms, using a more holistic ‘fecal volatilome’.

To date, analysis of volatiles from coprolites from different sources has not been attempted, and the possibility of utilizing VOCs as fecal biomarkers remains unexplored. Here, we conduct VOC analysis on pre-Clovis coprolites and their associated sediments from the archaeological site of Paisley Caves, OR, to test the hypothesis that *volatilomes can be used to distinguish between feces derived from carnivores, herbivores or humans* (**H1**). In addition, the hypothesis that *such volatilomes can distinguish between coprolites and closely associated non-fecal deposits where fecal material might penetrate***(H2)**, will also be tested.

### Sample information

**Figure 1:**
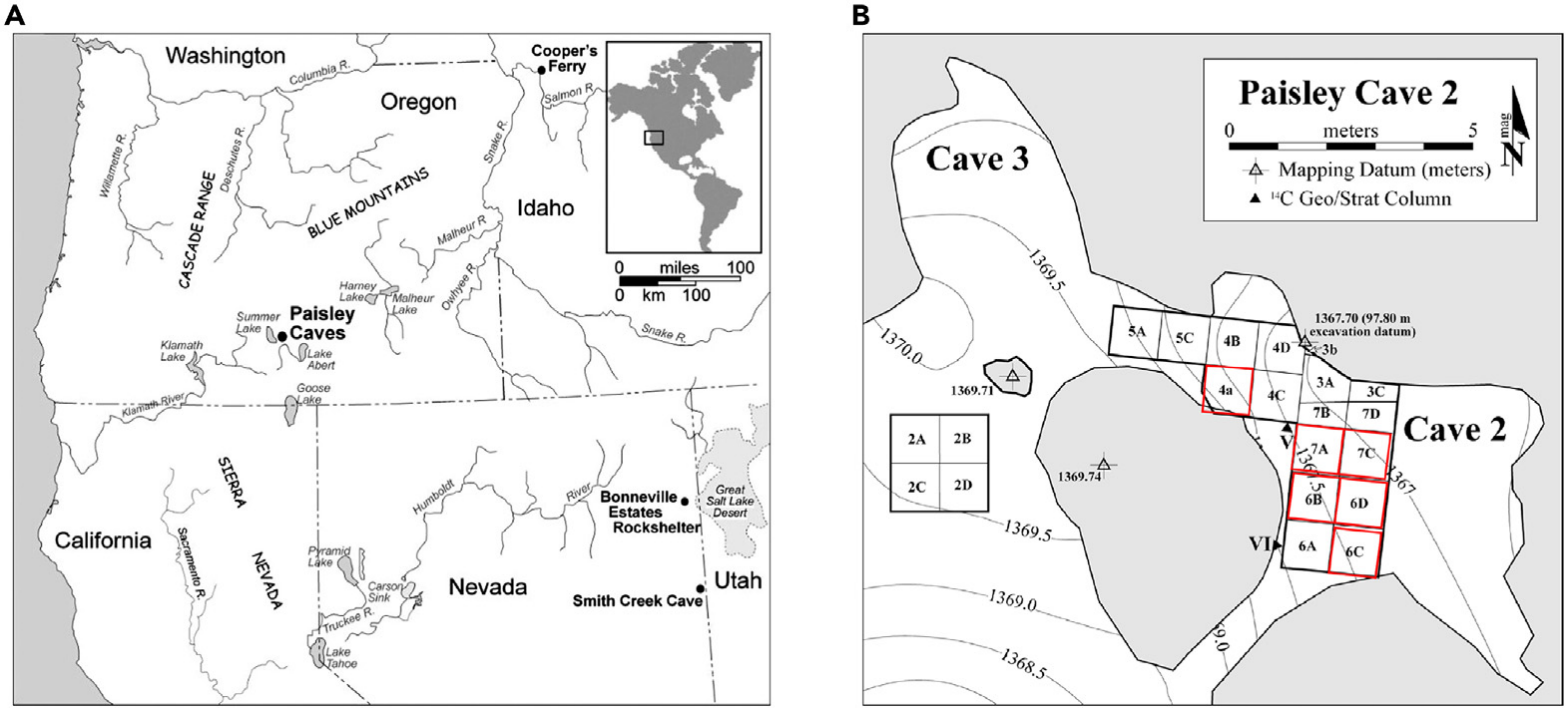
Sampling location of Paisley Caves (A) location of Paisley Caves in the US, (B) position of the quadrants within the cave where the samples were taken. Adapted from Jenkins et al. (2013).

Morphologically defined coprolites used in this study were collected from Cave 2 of the Paisley Caves complex (Figure 1). Sediments in Cave 2 consist of a sequence of aeolian, biogenic (e.g., bat guano and rat feces), volcaniclastic, and anthropogenic sediments.^15^ Stratum LU1 is a sandy gravel cave floor above the basalt bedrock. LU2 is a brown gravelly sandy loam as much as 30 cm thick overlying LU1. Both LU3 and LU5 are firm to hard fine sediments with abundant bat guano and woodrat midden debris. The stratigraphic unit locations of the coprolites are listed in Table 1. Background environmental controls (sediments) were collected underneath each coprolite during excavation.

**Table 1:** Classification of coprolites and sediments based on fecal lipid biomarkers

Sample ID	Paisley catalog number	Quadrant Location	Elevation (masl), context, and age	Inclusions	Phylogeny (Based on lipid biomarker)
23	1829-PC-2/4A-40-4a	4A	1366.08, LU3, N/A	Hair, bone	Carnivore
33	1896-PC-2/4A-55-1a	4A	1365.50, LU1, N/A	Hair, bone	Carnivore
39	1961-PC-2/7A-27-13a	7A	1365.55, LU2, N/A	Bone, plant material	N/A
45	1961-PC-2/7B-12-7a	7B	1366.12, LU3, N/A	Hair, round stone	Carnivore
47	1961-PC-2/7B-18-91a	7B	1365.68, LU3, N/A	Plant material	Herbivore
49	1961-PC-2/7B-20-9a	7B	1365.70, LU3, N/A	Hair	Carnivore
51	1961-PC-2/7C-10-5a	7C	1366.14, LU3, N/A	Hair, bone	N/A
63	1961-PC-2/7D-20-21a	7D	1365.71. LU3. N/A	Plant material	Herbivore
79	1829-PC-2/6B-28-1a	6B	1366.90, LU5, 4350 cal BP^a^	Plant material, angular stones	Herbivore
94	1896-PC-2/6C-49-5a	6C	1365.84, LU3, N/A	Hair, tooth and bone	Human
98	1896-PC-2/6D-51-12a	6D	1365.76, LU3, 12,515–12,087 cal BP^b^	Bone, hair, plant material, round stones	Human
101	1896-PC-2/6B-53-22a	6B	1365.67, LU3, 12,450 cal BP^a^	Hair, tooth, bone	Human

a Modeled age.^16^

b Direct date.^17^

Twelve coprolites and their associated sediments were analyzed in triplicate. A summary table of the lipid biomarker results can be found in the Supplementary Information (Table S1). Coprolites where source could not be determined using fecal biomarker analysis were removed from the following supervised statistical analysis.

## Results and discussion

### Utilizing VOCs as fecal biomarkers

**Figure 2:**
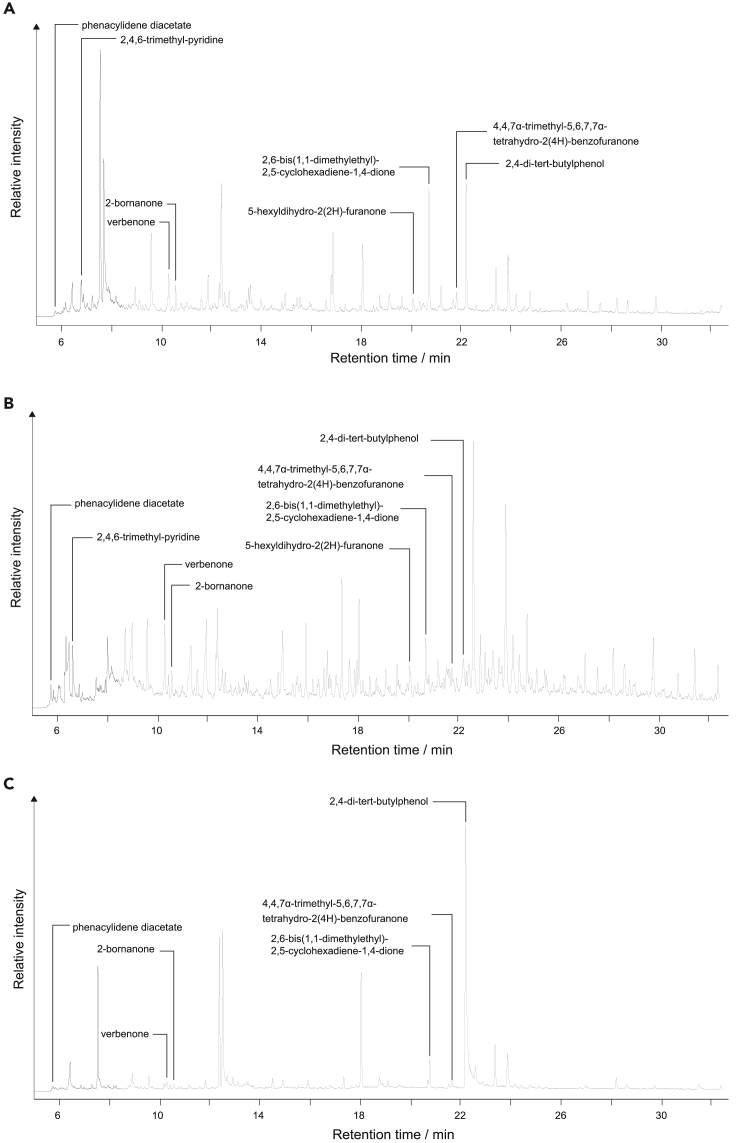
Partial gas chromatograms illustrating the typical distribution of discriminating volatile compounds (and others) (A) carnivore (S33), (B) human (S98), (C) herbivore (S63).

The headspace VOCs of 12 coprolites were analyzed by gas chromatography-mass spectrometry (GC-MS). The total ion current chromatograms (TICCs) of representative samples from each coprolite source are shown in Figure 2. After deconvolution and raw data configuration, up to 269 entities remained for further discrimination data analysis.

**Figure 3:**
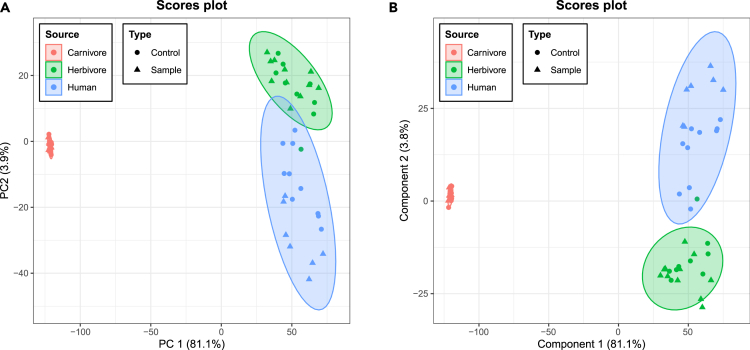
Clustering patterns of volatile profiles extracted from Paisley coprolites (A) PCA score plots for coprolites VOC profiles and (B) PLS-DA score plots for coprolites VOC profiles.

A PCA was initially conducted to show the PCA scores and clustering for samples (Figure 3A). As can be seen, coprolites from a particular source and the sediments associated with them cluster together, corresponding to the pre-determined lipid fecal biomarker analyses. Coprolites originating from carnivores can be clearly differentiated from those originating form herbivores and humans, indicating that the discriminatory VOCs between the coprolites could potentially be used to separate different coprolite sources. Even though herbivores and humans are more closely related along PC1, they show distinct clustering along PC2 and can therefore still be separated from one another. The unpaired T-test (p = 0.05) between each source (Figure S1) also supports that compared with human vs*.* herbivore, carnivore vs*.* human and carnivore versus herbivore have more entities that show both large fold change and statistical significance. The greater similarity between herbivores and humans may be because of a high proportion of plants in the diet of Paisley Caves occupants. Morphological studies identifying coprolite contents have shown that early occupants of Paisley Cave 2 clearly consumed a diverse diet where a high frequency of plant material was found to be consumed with all meals, and there were likely periods of time when diets comprised mostly plants.^18^^,^^17^ Fecal lipid biomarker analysis of these coprolites also found a high proportion of plant sterols (Table S1), confirming a substantial dietary input from plants. The results from the VOC analysis are consistent with those obtained from the analysis of fecal biomarkers, with coprolites clustering according to their assigned lipid profile. This supports **H1** in that it is possible to use VOCs distinguish between different fecal sources.

It is also noted that control sediments and samples within each group cannot be separated from each other. This is likely because of the presence of translocated discriminatory compounds in the sediments with degraded organic matter suggesting, unsurprisingly, that some leaching of low relative molecular mass and/or high polarity lipids from the coprolite into the surrounding soil has occurred. VOCs are relatively mobile in soils under varying pH and redox conditions.^19^ For example, VOCs are attenuated within leachate of landfill resulting from degradation and volatilization of organic deposits.^20^ Fatty acids can be soluble in water because of their polar nature, enabling leaching via ground waters. This is especially the case for low molecular weight fatty acids that have shorter, alkyl chains conferring less hydrophobicity. Water movement through the soil can also supply oxygen thereby accelerating the degradation of lipids^21^ producing degradation products that generally exhibit greater solubility in water. Parallel assessment of fecal biomarkers (5β-stanols and bile acids) shows no evidence of similar translocation, most likely because of their greater hydrophobicity retarding any movement arising from leaching. Therefore, it is only possible to differentiate between the VOCs of different coprolites when they are morphologically defined, thereby disproving **H2**.

Because PCA can only demonstrate the clustering patterns rather than distinguish discriminative compounds, a one-way analysis of variance (ANOVA) with a p value of 0.05 was applied to select the entities with significant differences between each sample group. Entities that have a p value of less than 0.05 are believed to have a significant difference.

PLS-DA was also used, as a more powerful supervised statistical method, to identify potential discriminating compounds that could characterize the source of coprolites. In both cases, a clear separation among different coprolite producers is observed. As shown in Figure 3B, PLS-DA reveals that the volatile profiles of coprolites are better separated between herbivore and human, enabling discrimination between sources. In addition, the cross-validation result shows that the PLS-DA model demonstrates highly distinguishable groups and high predictive ability (Q^2^ = 0.92, R^2^ = 0.96, Accuracy = 0.97). VOCs with VIP scores higher than 1 are considered of major importance and could be used for distinguishing coprolite source.

After statistical analyses, the identified entities have both VIP scores >1 and p values <0.05, indicating both high importance in discriminating coprolite sources and statistical significance, 8 compounds were tentatively identified (Table 2).

**Table 2:** List of identified volatile compounds for discriminating coprolite sample groups after selection of ANOVA test (p < 0.05) and PLS-DA (VIP >1)

Compound identification	Retention time (min)	VIP score	p value
phenacylidene diacetate	5.74	1.27	4.4E-21
2,4,6-trimethyl-pyridine	6.66	1.19	1.9E-12
4,6,6-trimethyl-bicyclo[3.1.1]hept-3-en-2-one (verbenone)	10.29	1.30	8.7E-17
2-bornanone (camphor)	10.56	1.21	3.6E-21
5-hexyldihydro-2(3H)-furanone	20.05	1.23	4.7E-21
2,6-di-*tert*-butyl-1,4-benzoquinone	20.69	1.07	1.2E-11
4,4,7α-trimethyl-5,6,7,7α-tetrahydro-2(4H)-benzofuranone	21.66	1.13	2.5E-20
2,4-di-*tert*-butylphenol	22.19	1.19	9.4E-20

### Compound identification and possible sources of discriminatory compounds

**Figure 4:**
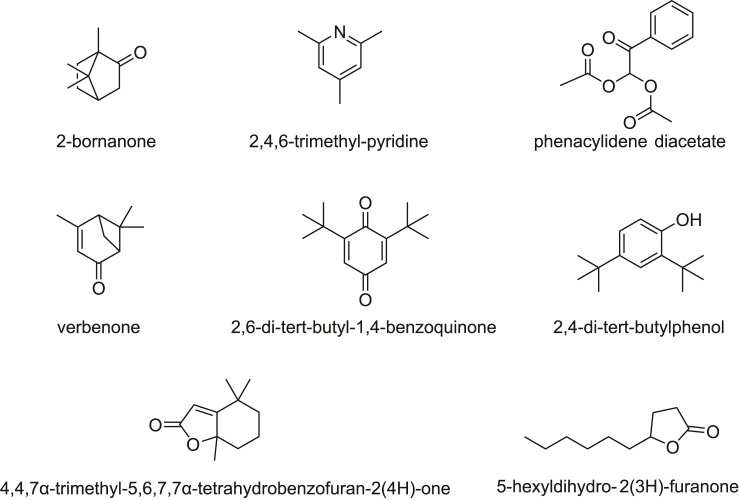
Structures of identified discriminative compounds

Structures of VOCs considered as potential discriminating compounds are shown in Figure 4. The identities of these compounds were further confirmed using authentic reference standards. All the retention times and mass spectra of these tentative compounds in coprolites correlate with those of the authentic reference compounds, indicating a high confidence in their identities.

The compounds identified were classified by the following functionality: cyclic/heterocyclic compounds alkane (1), ketones (5), phenol (1) and ester (1), and nitrogenous compounds (1). Most of the compounds are likely derived from dietary digestive residues because of their plant-derived origins. Table 3 shows the possible sources of these discriminative compounds. Previous studies of microfossils and bones from Paisley coprolites have suggested that occupants of Paisley Cave 2 had a highly variable diet including small mammals and insects, with plant material being consumed with all meals.^22^^,^^17^ Morphological identification of plant material in the coprolites yielded a range of plant fibers, seeds and pollen from various families such as sagebrush, amaranth, sunflower, mustard, carrot and a variety of grasses.^22^^,^^17^ Although certain discriminating VOCs identified in the coprolites can be linked to specific plant families, such as phenacylidene diacetate to the mustard family, it is difficult to identify the exact plant species using this approach. Furthermore, the VOCs potentially arising from beetle remains in the coprolites were found to parallel the beetle content of the coprolites. Apart from the dietary sources, the other substances detected from the VOC profiles of coprolites might be volatile metabolites arising from fatty acid degradation,^23^ or products of gastrointestinal microbial metabolism. Despite the many potential sources of discriminatory compounds identified in the coprolites, linking VOCs to a specific digestive function or process remains challenging as VOCs are secondary metabolites and there are multiple degradative changes that VOCs could undergo within the coprolites over time. In addition, the heating requirement of the extraction procedure does raise the possibility that that these compounds could be thermal degradation products of organics in the coprolite rather than extant VOCs. Nevertheless, the discrimination observed between coprolite sources and good matching with previous lipid identification results does support the notion that VOCs whether heat-generated or extant may be used to differentiate between coprolite sources thus, with work extending beyond Paisley applied across multiple archaeological contexts to better constrain the source(s) of the discriminatory compounds, VOCs possess a high potential as a rapid means for assessing the sources of coprolites.

**Table 3:** Potential origins of discriminative compounds

Compound	Verified with standards?	Potential origin of compound
2-bornanone	Yes	2-bornanone, also known as camphor, is a member of the class of cyclic monoterpenoids. Camphor as a VOC has been found in the volatiles from fecal shields of *Cassida denticollis* (a leaf beetle) as a defense from predators.^24^*Artemisia tridentata* (Great Basin big sagebrush) and *Tanacetum camphoratum* (camphor tansy) contain camphor^25^ and the former was and is still used by Indigenous people of western North America as a medicinal plant^26^; the latter can be poisonous to livestock although^27^ some do eat it, producing "unpleasant" tasting milk.^28^ This compound therefore most likely originates from the diet of a herbivore/human.
2,4,6-trimethyl-pyridine	Yes	2,4,6-trimethyl-pyridine has been identified as one of the VOCs from human urine,^29^ which is believed to result from the bacterial action within the gastrointestinal environment.^30^ This compound therefore likely originates from the human gut.
phenacylidene diacetate	No	Phenacylidene diacetate has been identified from the headspace of carbohydrate fragmentation compounds^31^ using SPME. It has been identified as one of the VOCs in the headspace of different cabbage cultivars^32^ and *Moringa oleifera*,^33^ however both plants are not native to North America.^34^
4,6,6-trimethyl-bicyclo[3.1.1]hept-3-en-2-one	No	4,6,6-trimethyl-bicyclo[3.1.1]hept-3-en-2-one,also known as verbenone, is a terpene that is found in a variety of plants, and is an oxidation product of α-pinene a constituent of Pinus spp.^35^ It has also been identified as an anti-aggregation pheromones for beetles including the mountain pine beetle (*Dendroctonus ponderosae*).^36^^,^^37^ Pine pollen and beetle skeletons were also found in Paisley coprolites,^22^^,^^17^ indicating unintentional consumption by humans and a likely source of this compound.
2,6-di-*tert*-butyl-1,4-benzoquinone	Yes	2,6-di-*tert*-butyl-1,4-benzoquinone (BHT-Q) is a monocyclic monoterpenoid. It has been found in headspace of human excreta^38^ and involved in many biochemical processes such as lipid and fatty acid metabolism.^39^ It has also been found in Celastrus,^40^ which is a genus found in the eastern North America^34^ and commonly used by Native Americans as medicine.^41^
2,4-di-*tert*-butylphenol	Yes	2,4-di-*tert*-butylphenol (2,4 DTBP) belongs to the chemical class of phenols. It is a common natural product, which can be found as a secondary metabolite in 16 species of bacteria in 10 families.^42^ It can be detected in human feces (Yan et al., 2020). 2,4-DTBP was also reported in different groups of plants, such as liverwort and ferns.^42^*Chamaebatiaria millefollium* is a fragrant fern bush found in the Great Basin, and the leaves were possible food candidates, which were browsed by mule deer, sheep, and goats.^43^
4,4,7α-trimethyl-5,6,7,7α-tetrahydro-2(4H)-benzofuranone	No	This compound, also called dihydroactinidiolide, is a terpene lactone, and is an oxidative degradation product of carotenoids. As a natural product, it is known to be a pheromone in insects^44^ and in mammals such as cat and red fox.^45^ Also it has been isolated from various of plant leaves and fruits,^44^ such as Prosopis spp.^46^^,^^47^ Specifically, *Prosopis glandulosa* was a native plant in the southernmost Great Basin area,^48^ which was food source to a variety of herbivore animals^49^ and humans.^50^
5-hexyldihydro-2(3H)-furanone	Yes	Natural occurrence of 5-hexyldihydro-2(3H)-furanone has been reported from plant species called *Ammodaucus leucotrichus* belonging to the Apiaceae (parsley) family.^51^ There is no direct evidence that this plant is native to North America, however in the Great Basin area, Apiaceae family is very common,^52^ therefore it is likely that this compound originates from the diet of a herbivore/human.

### Limitations of the study

A limitation of the study is that the heating requirement during the extraction procedure does raise the possibility that that compounds extracted are thermal degradation products of organics in the coprolite rather than extant VOCs. However, the discrimination observed between coprolites from different sources and good matching with previous lipid identification results would suggest that VOCs irrespective of being heat-generated or extant are a means of differentiating between coprolite sources.

## STAR★Methods

### Key resources table

**Table:** 

REAGENT or RESOURCE	SOURCE	IDENTIFIER
**Chemicals, peptides, and recombinant proteins**		

ethyl acetate (HPLC grade)	Rathburn, UK	CAS: 141-78-6
2-bornanone	SigmaAldrich	CAS: 464-49-3
2,4,6-trimethyl-pyridine	SigmaAldrich	CAS: 108-75-8
2,4-dimethyl-benzaldehyde	SigmaAldrich	CAS: 15764-16-6
2,6-bis(1,1-dimethylethyl)-2,5-cyclohexadiene-1,4-dione	SigmaAldrich	CAS: 50348-20-4
2,4-di-tert-butylphenol	SigmaAldrich	CAS: 96-76-4
5-hexyldihydro-2(3H)-furanone	SigmaAldrich	CAS: 7011-83-8

**Software and algorithms**		

MassHunter Workstation Profinder	Agilent Technologies	B.08.00
Mass Profiler Professional	Agilent Technologies	B14.5
R	R core team	v4.0.2

### Resource availability

#### Lead contact

Further information and requests for resources and reagents should be directed to and will be fulfilled by the lead contact, Dr Ian D. Bull (ian.d.bull@bristol.ac.uk)

#### Materials availability

This study did not generate new unique reagents.

### Method details

#### SPME volatile extraction

The experimental design was modified from that of Brown et al*.*^14^ Coprolites and sediments were crushed using a mortar and pestle and then passed through a 2 mm sieve. Approximately 0.05 g of each sample was placed into a 10 mL headspace vial with PTFE/silicone septa lid (Fisher Scientific, UK) and five drops of double distilled water were added to accelerate the release of VOCs.^53^ The sample was then placed in a heating block and, maintained at a temperature of 60°C for 1 h. After sample incubation, a solid-phase microextraction (SPME) fibre (50/30 μm DVB/CAR/PDMS; Supelco, Bellefonte, USA) in a manual SPME holder (Supelco, USA) was used to sample the head-space air above faeces for 20 min. The SPME fibre was conditioned in injector of the gas chromatograph (GC) at 250°C for at least one hour prior to each sampling event, thereby ensuring that no interfering peaks were obtained. The fibre was then retracted and immediately (<1 min) manually injected into the injection port of a GC/Q-TOFMS for characterisation.

#### Volatile reference mixture preparation

Identities of all VOCs considered as potential discriminating compounds were confirmed using authentic reference standards. Volatile reference solutions were created from a combination of individual standards, including: (+)-2-bornanone, 2,4,6-trimethyl-pyridine, 2,4-dimethyl-benzaldehyde, 2,6-bis(1,1-dimethylethyl)-2,5-cyclohexadiene-1,4-dione, 2,4-di-tert-butylphenol, and 5-hexyldihydro-2(3H)-furanone, each at a concentration of 400 μg mL^–1^. The solvent used for dilution was HPLC-grade ethyl acetate (Rathburn, UK). The final volatile reference mixture was prepared by adding 100 μL of each standard solution to the headspace vial, followed by SPME extraction at room temperature for 20 min. The extracted standards were then manually injected into and characterised by GC/Q-TOFMS.

#### Instrumental analyses

GC-MS analyses were performed using an Agilent 7890B GC coupled to a 7200B accurate mass quadrupole time-of flight mass spectrometer (GC/Q-TOFMS). VOCs were introduced *via* a multimode injector maintained at 250°C, operating in split mode (1:10 split ratio). The analytical column had the dimensions 50 m × 0.32 mm × 0.17 μm with a 100% dimethylpolysiloxane stationary phase (Agilent J&W HP-1) with a helium carrier gas flow of 2 mL min^–1^. The oven temperature was programmed to increase from 60°C to 250°Cat 4°Cmin^–1^. The transfer line and source temperatures were set to 300°C and 230°C, respectively. The mass spectrometer was operated in EI ionization (70 eV) mode, and data were acquired across a range of *m/z* 25-400.

#### Data processing

Raw datasets were imported to MassHunter Workstation Profinder B.08.00 (Agilent Technologies, USA) for deconvolution and peak alignment. The algorithm “Batch Molecular Recursive Feature Extraction for Small Molecules” was applied to enable increased filtering parameters for chromatogram deconvolution, peak alignment, and feature extraction. The raw data were configured to a three-dimensional dataset as [mass × retention time × intensities] based on the chromatographic and spectrum information. The filtering parameters were: retention time range (5-40 min), retention time tolerance (0.05 min), *m/z* range (20-300), peak filters (500), ion count threshold (30), absolute height (10000 counts) and EIC extraction range.

After configuration, filtered data were imported to Mass Profiler Professional (MPP) version B14.5 (Agilent Technologies, USA) for data treatment. All of the ‘entity’ lists were then exported from MPP to R v4.0.2^54^ for statistical analysis and visualisation with packages MetaboAnalystR^55^ and ggplot 2.^56^

#### Statistical analysis

For data treatment, data transformation was set as a log2 transformation and mean-centred baselining to make a better comparison between samples. In addition, the following data filters were applied to select compounds that can represent each individual dataset: frequency filter (entities present in >90% samples in at least one sample group), variability filter (coefficient variable <25%), fold change (10) for selecting only the main discriminant compounds between samples. Finally, a one-way analysis of variance (ANOVA) with a *p* value of 0.05 was applied to select the entities with significant differences between each sample group.

Principle components analysis (PCA) was used to observe the clustering patterns of samples. PCA is an unsupervised method that shows natural clustering trends by reducing multidimensional data to a few principle components, revealing differences between measurements in its scores if those differences are major contributors to the total variability.^57^ Differences among clusters along the PC1 axis capture the most variations in the dataset and the PC2 axis captures the second greatest degree of variation. The lists of discriminating entities were extracted by comparison between each source category. Pair-wise comparisons were followed by Tukey’s HSD test to isolate entities that were different between species and Benjamini Hochberg FDR for multiple testing corrections.

Partial least-squares discriminant analysis (PLS-DA) was also used to identify potential discriminating compounds that could characterise sources of coprolites. PLS-DA is a classification method based on the regression extension of PCA. Unlike PCA which could not identify potential discriminating components, PLS-DA is a supervised approach which could predict variation of spectral features.^58^ Therefore, coprolites where source could not be determined using lipid biomarker analysis were removed from PLS-DA analysis.

To test if the model was robust and well-fit, a 10-fold cross validation method was used to indicate the sensitivity and predictive ability of the model. R^2^ provides a measure of model fitness to the original data, and Q^2^ is an estimate of the predictive ability of the variation, good predictions will tend to have both high R^2^ and ^2^^,^^59^^,^^60^ Moreover, the variable importance in projection (VIP) scores were calculated to estimate the importance of each component in predicting coprolite phylogeny based on the PLS-DA model. Components with VIP scores higher than 1 were considered of high importance, which are good indicators for discriminating the coprolite source identity. Components with VIP > 1 and *p* < 0.05 were therefore considered as discriminating compounds.

Finally, entities responsible for the greatest variance were tentatively identified using their determined accurate mass and matching with the NIST 14.0 library entries and literature data. To eliminate misidentification, peak inspection was conducted manually to assess each candidate spectrum.

## Data Availability

• Raw instrument data reported in this paper will be shared by the lead contact upon request, a table of entities used to construct the PCA and PLS-DA plots is included in the Supplementary Data (Table S2: Entity list used to construct PCA and PLS-DA analysis). • This paper does not report original code. • Any additional information required to reanalyse the data reported in this paper is available from the lead contact upon request.

## Supplemental information

Document S1. Figures S1 and S2

Table S1. Fecal lipid biomarker analysis of coprolites from Paisley Cave 2, related to Figure 3

Table S2. Entity list used to construct PCA and PLS-DA analysis, related to STAR methods
