# Supplementary material for: Volatile organic compounds (VOCs) as a rapid means for assessing the source of coprolites
Source: iScience. 2023 May 4;26(6):106806. doi: 10.1016/j.isci.2023.106806 (PMC10225897; doi:10.1016/j.isci.2023.106806)
Supplement: Document S1. Figures S1 and S2 [file mmc1.pdf]

iScience, Volume *26*

Supplemental information

Volatile organic compounds (VOCs) as a rapid means for assessing the source of coprolites

Wanyue Zhao, Helen L. Whelton, John C. Blong, Lisa-Marie Shillito, Dennis L. Jenkins, and Ian D. Bull

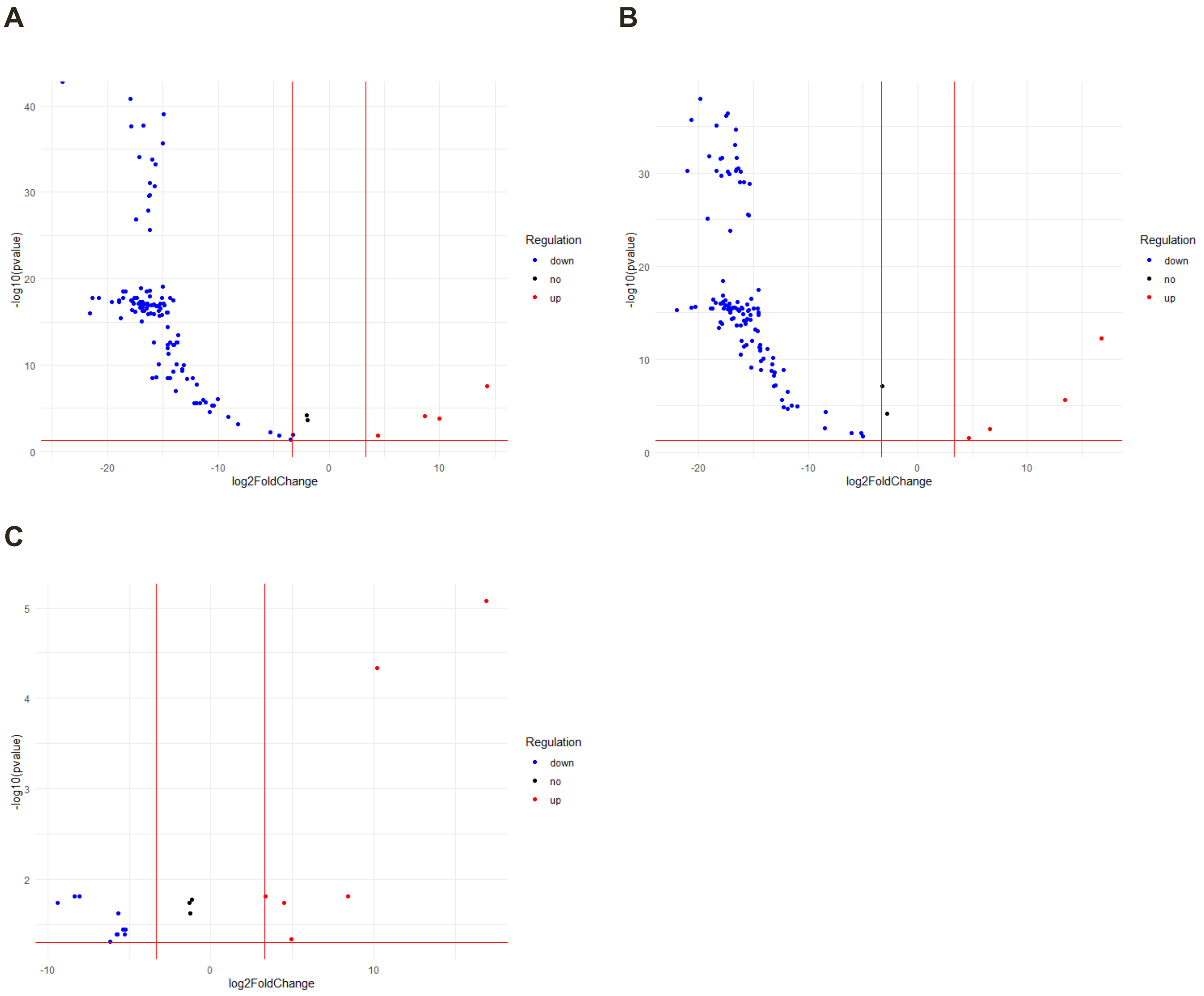

Figure S1: Volcano plots of -log10 (*p* value) against fold change, representing the compound difference between (A) carnivore and herbivore, (B) carnivore and human, (C) human and herbivore, related to STAR method. The blue dots indicate compounds of interest that display both large magnitude fold changes and high statistical significance, black dots indicate compounds that are not significant, red dots indicate compounds that have high statistical significance but low magnitude fold changes. The red lines display the threshold where $p = 0.05$ and fold change cut-off = 10.

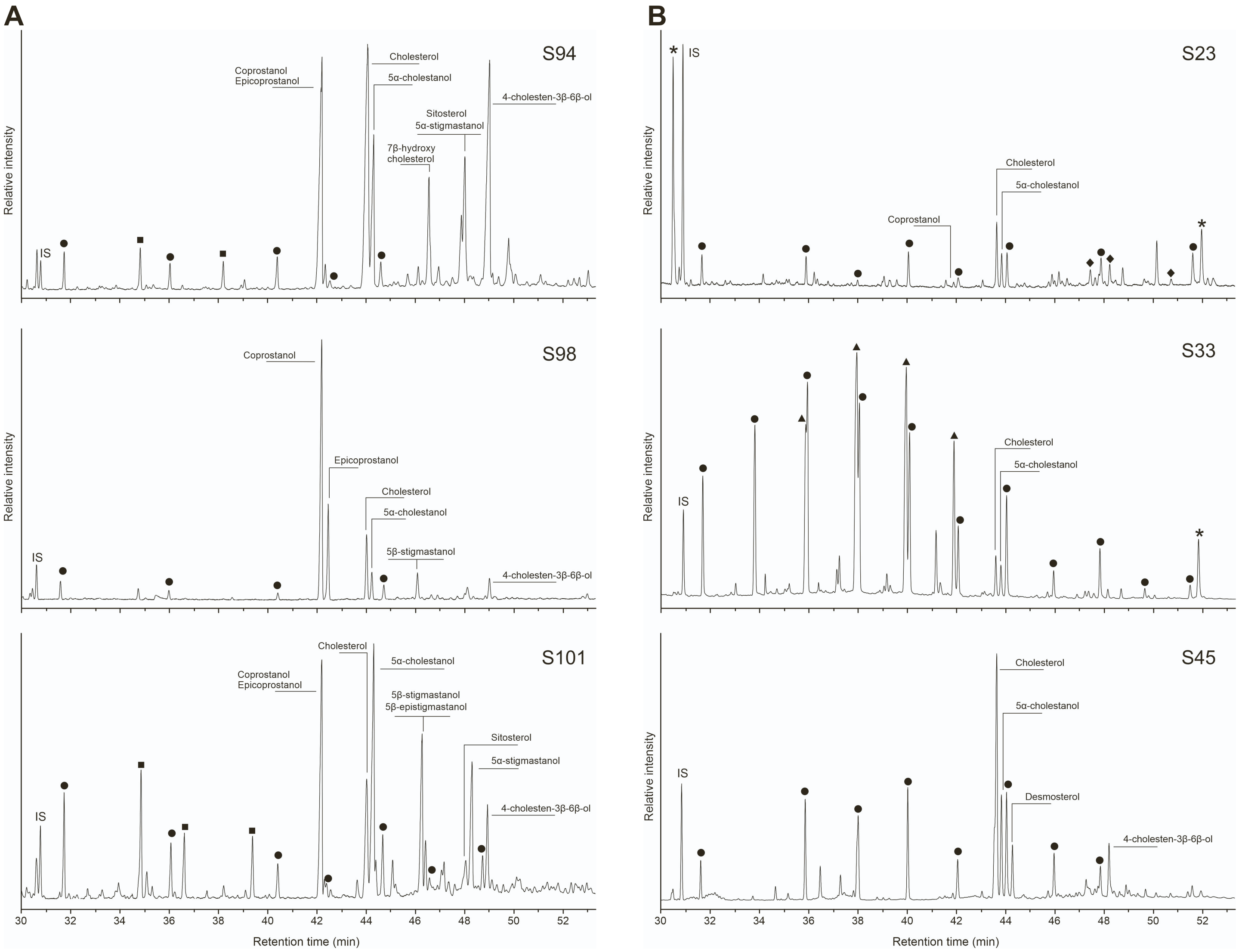

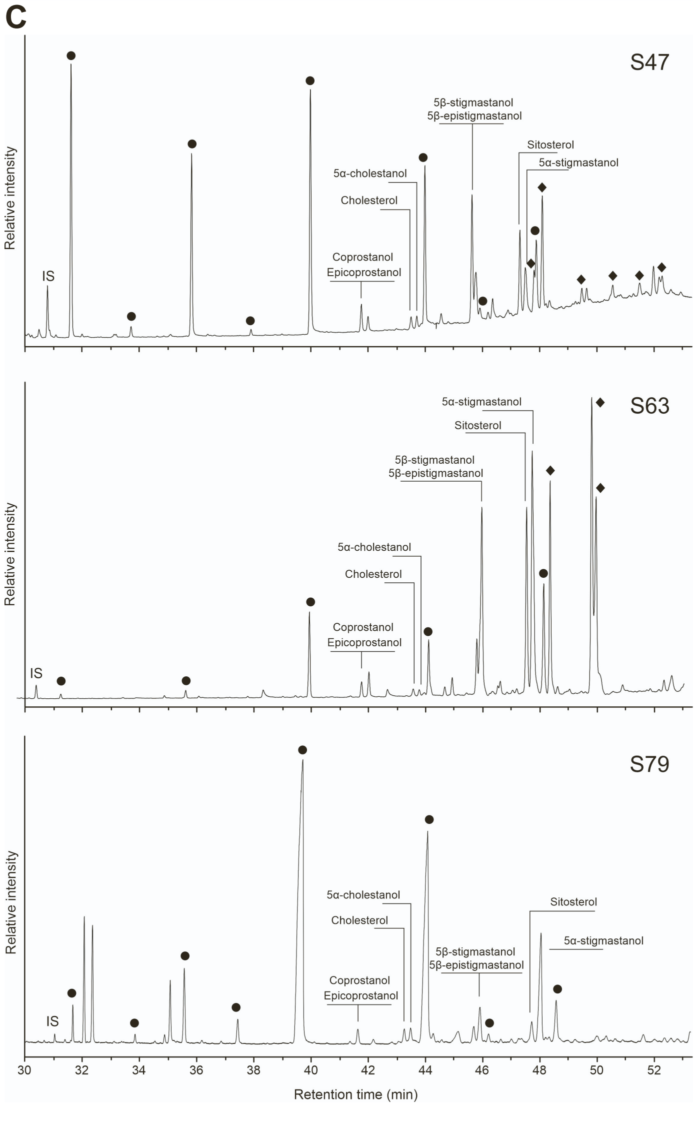

Figure S2: Partial gas chromatograms illustrating the distribution of steroid compounds in coprolites identified as (A) human, (B) carnivore, or (C) herbivore, related to Figure 3. Where ● denotes *n*-alcohols of carbon chain length C_{22} to C_{30}, ▲ denotes 1,2-diols, ■ alkylglycerols, ♦ denotes triterpenoids all as TMS derivatives. IS denotes added internal standards: preg-5-en-3β-ol is the sterol standard and * denotes contamination (plasticiser).
